# Editorial: Benchmarking 3D-Models of Root Growth, Architecture and Functioning

**DOI:** 10.3389/fpls.2022.902587

**Published:** 2022-05-26

**Authors:** Andrea Schnepf, Daniel Leitner, Gernot Bodner, Mathieu Javaux

**Affiliations:** ^1^Institute of Bio-Geosciences (IBG-3, Agrosphere), Forschungszentrum Jülich GmbH, Jülich, Germany; ^2^Simulationswerkstatt – Services in Computational Sciences, Linz, Austria; ^3^Department of Crop Sciences, Division of Agronomy, University of Natural Resources and Life Sciences BOKU Vienna, Tulln an der Donau, Austria; ^4^Earth and Life Institute, Université Catholique de Louvain, Ottignies-Louvain-la-Neuve, Belgium

**Keywords:** root architecture modeling, benchmarking, root hydrology, plant-soil interaction, mathematical modeling, simulation

Three-dimensional models of root system development and functioning have evolved as important tools that aid designing agricultural management schemes for improved resource use efficiency and selecting root traits for optimizing plant performance in specific environments (Benes et al., 2020). For their reliable application, benchmarking of such so-called functional-structural root architecture models (FSRM) is urgently needed. Similar relevant benchmarking initiatives have been performed for crop models (AgMIP), reactive transport models (Steefel et al., 2015), or models of water flow and solute transport in soils (Vanderborght et al., 2005). FSRMs generally solve flow and transport equations in the soil and in root system, and couple them via different approaches. Differences between different models' outputs might arise from differences in mathematical formulation of the processes and their coupling, in the numerical scheme, but also from coding errors. Consequently, potential errors might propagate into the plant and soil interaction simulations relying on an accurate simulation of root architecture development for describing root water and solute uptake processes. This Research Topic set out to shed some light on the extent of potential uncertainty due to these different factors.

Benchmarking is an emerging procedure to measure performance of models against a set of defined standards (Luo et al., 2012). In this issue, Schnepf et al. announced a “*Call for Participation: Collaborative Benchmarking of Functional-Structural Root Architecture Models. The Case of Root Water Uptake*”. They designed benchmark problems for root growth models, soil water flow models, root water flow models, and for water flow in the coupled soil-root system. All the benchmarks and corresponding reference solutions were published in the form of Jupyter Notebooks on the GitHub repository https://github.com/RSA-benchmarks/collaborative-comparison. Several groups that develop such functional-structural root architecture models have contributed with their solutions to the benchmark problems on this GitHub repository, and it may provide orientation for future model developments as well. The benchmarks follow a multi-step approach with growing level of complexity regarding both the number of processes accounted for and the dimension of the system. The first set of benchmarks is about individual modules only. The scenarios are simple, potentially solvable with analytical solutions, and the goal is to build trust in accuracy of the individual models. The second set of benchmarks is about the fully coupled models, with a focus on comparison of numerical representation of agreed-upon equations and process representations. In the third set of benchmarks, models do not have to have the same process representations. Evaluations of those are only possible against available data sets and by comparing the different model outputs.

In this issue, Khare et al. further extended the benchmark problem Schnepf et al. which is about root water uptake from a drying soil. They showed in a grid convergence study that the additional resistance to water flow toward the root surface caused by a dry rhizosphere must be considered for dry soil or else root water uptake is significantly overestimated. Simulations were performed with dumux-rosi. Solutions to the problem of dry rhizosphere are presented in Khare et al., but also in (Schröder et al., 2009a,b; Beudez et al., 2013; Mai et al., 2019; Koch et al., 2021). All of those solutions include a way to determine sub-resolution scale (with respect to the soil grid) rhizosphere water potential gradients in a computationally efficient way. The alternative is grid refinement but this comes at high computational costs as also discussed in Khare et al..

As part of this issue, soil compaction due to agricultural traffic and resulting mechanical and hydric stresses and their effect on root water uptake were simulated by de Moraes et al. using CRootBox (Schnepf et al., 2018) coupled with a 1-dimension soil water flow model. The model simulations could elucidate the feedback between root function and local soil stresses at the field scale for a Brazilian Oxisol. Here, the reference is not a mathematical reference solution but a reference data set. For field-scale simulations, FSRMs are often coupled to models that have 1-dimensional soil modules, e.g., crop models (Wu et al., 2015; Seidel et al., 2022). From a known 3D root hydraulic architecture, 1-dimensional sink terms can be derived (e.g. as shown in Vanderborght et al., 2021).

Model simulations may elucidate the contributions of different root types to overall plant nutrient uptake. Using OpenSimRoot (Postma et al., 2017), Gonzalez et al. indicated in this issue that nodal roots contribute most to P uptake by rice plants, followed by L-type lateral roots, S-type laterals and root hairs, but these strategies have different carbon costs. Implications for improving adaption to P deficiency in rice breeding are discussed. These results have to be also seen in light of the respective soil P and water content (De Bauw et al., 2020).

The longitudinal pattern of root aerenchyma formation modeled by the Ti-Gompertz model helped to deeply understand the relationship between the anatomical traits and physiological function in rice adventitious roots (Chen et al., as part of this issue). Such data will help to further develop models that include information on the root anatomy such as MECHA (Heymans et al., 2021) and GRANAR (Heymans et al., 2019).

Through this Research Topic, we continue to provide the opportunity to participate in the development and application of suitable benchmarks. This exercise allows us to point out sources of inaccuracies, knowledge gaps and to pin-point current challenges in mathematical model development of FSRM's.

## Author Contributions

All authors listed have made a substantial, direct, and intellectual contribution to the work and approved it for publication.

## Conflict of Interest

The authors declare that the research was conducted in the absence of any commercial or financial relationships that could be construed as a potential conflict of interest.

## Publisher's Note

All claims expressed in this article are solely those of the authors and do not necessarily represent those of their affiliated organizations, or those of the publisher, the editors and the reviewers. Any product that may be evaluated in this article, or claim that may be made by its manufacturer, is not guaranteed or endorsed by the publisher.
